# Routine, ensemble characterisation of electrophoretic mobility in high and saturated ionic dispersions

**DOI:** 10.1038/s41598-020-61624-9

**Published:** 2020-03-13

**Authors:** Jake Austin, Diogo Fernandes, Matthew J. A. Ruszala, Natalie Hill, Jason Corbett

**Affiliations:** Nanotechnology group of Malvern Panalytical Ltd., Grovewood Road, Malvern, WR14 1XZ United Kingdom

**Keywords:** Environmental monitoring, Techniques and instrumentation

## Abstract

With the industrialisation of nanoparticle manufacture, the pervasive incursion of nanoparticles into the environment, the need to characterise nano-scale pharmaceuticals and living systems in replicated *in vivo* conditions, the continuing development of new theories to describe the electro-kinetic behaviour of nano-particles in representative ionic strengths and numerous other applications, there is an urgent requirement to provide simple and effective experimental tools to validate these models and explore new systems. Micro-electrophoresis implemented with a diffusion barrier, which isolates the dispersed phase from the electrode surface, is demonstrated as enabling such measurements for the first time, preventing the catastrophic outgassing, precipitation and sample degradation observed when the dispersed phase is in close proximity to the electrode surface. Using a measurement of a few minute’s duration in a standard laboratory light scattering instrument we reproduce the theoretically predicted phenomena of asymptotic, non-zero electrophoretic mobility with increasing ionic strength, the cationic Hofmeister series dependency, charge inversion and a continuously decreasing variation in mobility with *pH* as molarity increases. Standard operating procedures are developed and included to encourage further work.

## Introduction

The action and interaction of colloidal or nano-scaled biological nano- or micro-scaled particles dispersed in a continuous liquid phase is of significant interest in a range of applications^1–8^ and in particular there is urgent interest in the behaviour of bio-particles in living systems, such as proteins and lipids^9–12^, the targeted delivery of otherwise toxic pharmaceuticals by encapsulation to avoid a negative response from the immune system^13–16^, the behaviour of naturally occurring colloids^17–19^ and the incursion and removal of manufactured nano-scale to micro-scale particles into the environment^20–24^ and thence, also into living organisms^25–29^.

A key indicator of the stability of colloidal and protein dispersions is the particle zeta potential^30^. On immersion into aqueous media, charged ionic species gather at the particle surface, creating a complex layer of charges known as the electrical double layer (EDL), comprising of a stationary layer of species adsorbed, chemically bound, to the particle surface and around this inner layer, a diffuse layer attracted via Coulombic interactions, which acts to minimise the total energy in the system by screening the first layer. The electric potential at the hydrodynamic slipping plane within the diffuse layer is defined as the zeta potential and is an indicator of how the dispersed phase is to interact with itself and its surroundings over time. For instance, DLVO theory^30^ describes how dispersed particles interact with each other as their EDL’s overlap with an attractive Van der Waals and repulsive Coulombic potential interaction assumed, whose sum creates a pair of sequentially deeper potential wells as the particles approach one another. Within the outer (secondary) well the particles are loosely agglomerated and may be re-dispersed, however, if the equilibrium state has sufficient kinetic energy the particles may reach the inner (primary) well, where the attractive Van der Waals forces overcome the Coulombic force at short distances. This process is irreversible and *en masse* the dispersion will tend to aggregate over time.

The classical Gouy-Chapman model^30^ describes the charge distribution around an *individual particle*, when dispersed in a fluid, as an equilibrium between the thermal motion of the bulk ions using Maxwell-Boltzmann statistics and the electrostatic field created by the surface charge and there are a number of models that describe the extension of this model to real systems^30–33^. In the classical model, the judicious alteration of the *pH* of the continuous phase can be used to change the exposed charge at the particle surface and, subsequently, the zeta potential can be engineered to be either high in magnitude if aggregation is undesirable, or low in magnitude, if it is to be encouraged. Additionally, the dispersion itself can be changed in composition or concentration, altering the Debye length^30^, which describes the range of the Coulombic interaction from the particle surface. As the ionic concentration of the dispersion increases the ‘double layer is suppressed’ meaning that more and more charge in the vicinity of the stationary part of the double layer reduces the Debye length in a screening action. However, many of the applications where the characterisation of zeta potential is important, including many of those noted above, need to be conducted at ionic strengths that far exceed those assumed by the Gouy-Chapman model. The classical model fails at and approaching ionic saturation, because of: -

1. steric hindrance between ions^34,35^,
2. the solvation shell around an ion lowers the local dielectric permittivity causing an additional dielectrophoretic force in an applied field gradient^36,37^,
3. changes in permittivity due to the ordering of water molecules in the interfacial layer^38^,
4. the possibility of a zero charge, but non-zero EDL, due to the ion specific penetration of a structured water layer at the particle surface^12,38^,
5. the Debye length becomes smaller than the Bjerrum length over which ion-ion correlations become significant^39^,
6. ion-ion pair correlations and ion size resulting in over screening of charged surfaces and charge reversal^36,39^,
7. the Debye length becomes comparable to the ion size: ions ‘collapse onto the particle surface’^38^
8. and the detailed structure of the EDL at high salt depends on the identity of the ions present^36,39^.

Further still, Storey & Bazant^36^ note that ‘…any attempt to develop and modify continuum models for molecular scale phenomena is fundamentally limited…our goal is to develop and *test* [new] models…to facilitate a better understanding of electro-kinetics in macroscale experiments.’ Dukhin, Dukhin & Goetz^38^, state, ‘It is clearly very important to *verify* the existence of …zero charge double layers [a consequence of high ionic strength systems] and develop methods for its study…’ and Grainger & Castner^40^ note in their review of nanomaterial characterisation for the biomedical sciences, that ‘…[the] current literature is replete with many examples of…nanoparticles with little emphasis placed on reporting rigorous surface analysis or characterisation’. So, with a pressing need to address the applications noted above and the still developing nature of the underlying theory of saturated zeta potential measurements, it is clear that a simple, reproducible experimental platform which can measure electro-kinetic behaviour in high and saturated salt solutions is urgently required.

Saturated zeta potential measurements have been reported using an ultrasound technique^40^ but in prohibitively large volumes for valuable manufactured or field-collected samples. Imaging velocimetry under a microscope has been reported^41^ but this technique measures small numbers of micron-scaled particles yielding a statistically poor sample from the parent population and missing the nano-scale completely. Further still, in order to detect the residual electrophoretic mobility in high molarities from which zeta potential is calculated and which, as we report in this work, tend to be rather low in magnitude, a potential difference across the cell must be applied for periods long enough that the images of the particles explore a region of the detector plane larger than the Rayleigh resolution of the imaging microscope. At high ionic strength this causes significant Joule heating thereby increasing the uncertainty in the measured electrophoretic mobility due to both the uncertain change in viscosity of the continuous phase, but also, possibly, by altering the electrophoretic mobility of a thermally sensitive dispersed phase, such as proteinaceous samples, for instance. Worse still, the titration of *pH* over an arbitrary range is a staple component of the electrochemist’s toolkit and long timescale field applications will create an electro-osmotic flow along the walls of the microscope slide that responds to the prevailing conditions (ionic strength and *pH*, in this work) of the continuous phase in the applied electric field. This may systematically increase the uncertainty in the electrophoretic mobility further still. Microelectrophoresis measurements have been demonstrated for ionic conditions up to 1.0 M^42^ and super-resolving microscopes may become more affordable in future but the science and application of very high and saturated ensemble, ionic systems and the imperative to understand them is maturing now.

**Figure 1:**
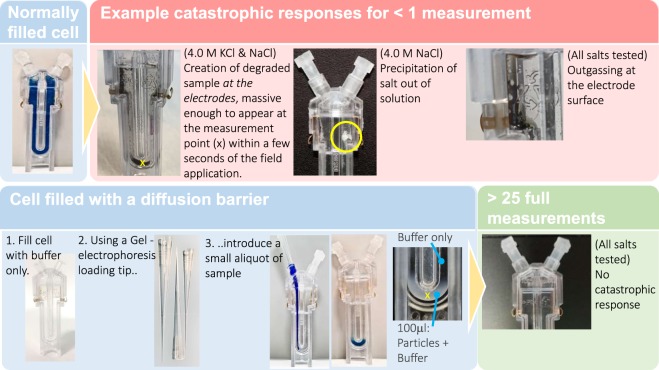
*Top row* – a microelectrophoresis cell filled completely with dispersant and dispersed phase (200 nm polystyrene beads) with associated examples of the resulting failure modes observed in <1 measurement, which consists of an applied electric potential of a ±10 V square wave at 10 Hz, for <20 s, with an effective distance between electrodes, along the length of the capillary, of ~50 mm. *Bottom row* – the diffusion barrier method, placing a small sample of dispersed phase in buffer only in the vicinity of the optical detection volume (x) of the instrument in a cell otherwise filled with buffer only. Electrical contact between the dispersed phase and the electrode is maintained through the buffer, but with the dispersed phase physically separated from the electrode surface. The absence of the dispersed phase at the electrodes is observed to prevent the catastrophic events in the top row for all salts tested, allowing the measurement to proceed.

Micro-electrophoresis is most commonly implemented in laboratory instrumentation using a closed capillary cell, with electrode chambers at each end: a disposable example of which (DTS0170/80, Malvern Panalytical Ltd, UK) is shown in Fig. 1.

With reference to Methods, below & Fig. 1 (*top-row*), if the cell is filled entirely with dispersed nanoparticles in a high ionic strength buffer and an electrical potential applied along the capillary commensurate with an acceptable signal to noise in the electrophoretic motion of the particles using the PALS measurement, then catastrophic effects such as the creation of large aggregated particle size fractions and outgassing have been observed for all salts tested. The precipitation of salt out of solution was also observed, however, this occurred only intermittently and only for NaCl, despite significant efforts to ensure that only high grade chemicals were used and the cells scrupulously cleaned and rinsed beforehand.

The diffusion barrier method is shown in Fig. 1 (*bottom-row*). The cell is filled with buffer with an admixture of buffer and the dispersed phase then added only to the optical detection volume, x at the base of the capillary, remote from the electrode surfaces. This technique was originally developed to minimise valuable sample volumes^43^, however, *we also find that the isolation of the dispersed phase from the electrode surface by filling the cell with a diffusion barrier prevents the catastrophic response in high ionic strength dispersions and allows the measurement to proceed*. A cell is shown (*bottom-right*) after greater than 25 such measurements, with a total measurement time of approximately 300 s, under identical conditions of sample preparation and instrument settings as the top row but with the cell loaded using a diffusion barrier. No catastrophic response occurs, and electrophoretic characterisation is able to proceed.

**Figure 2:**
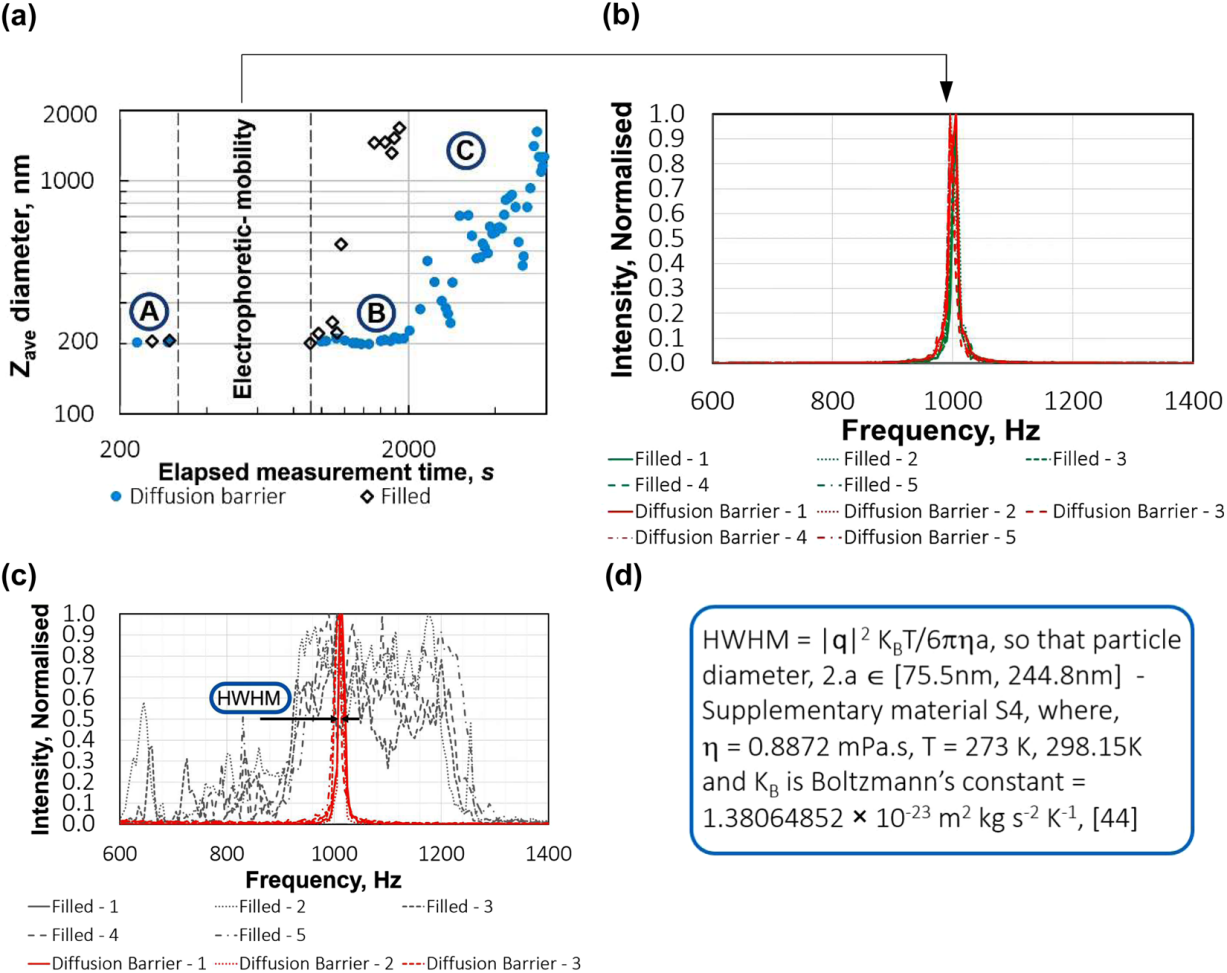
(**a**) DLS *Z*_*ave*_ (Cumulants^45^) size for 200 nm polystyrene in 200 mM KCl at pH 7.0, against time, (**b**) frequency distribution of 200 nm polystyrene in 200 mM KCl during electrophoretic mobility measurement for diffusion barrier and fully filled cases, (**c**) the frequency distribution of the 4.0 M measurements for the fully filled and diffusion barrier cases. Note that all frequency spectra are not centred at 0 kHz. This is a deliberate instrumental effect whereby the signal is modulated optically at 1 kHz to shift the sample spectrum and avoid background vibration from the environment of the instrument, such as footsteps, air-conditioning vibration, etc. Additionally, both sides of the distribution are accessible, which would not be the case for spectra centred at 0 Hz^45^, (**d**) particle size estimate from the half-width – half-maximum (HWHM) of the frequency spectrum.

In more detail, Fig. 2(a) shows the reported hydrodynamic size (the cumulants *Z*_*Ave*_ value^44^) of 200 nm polystyrene nano-spheres dispersed in 200 mM KCl, before and after the electrophoretic mobility measurement. Prior to and just after the electric field the reported hydrodynamic size is 206 nm ± 3 nm, well within the hydrodynamic range reported by the manufacturer, for the both the fully filled and diffusion barrier cases. However, within approximately 150 s of the end of the electro-phoresis measurement the reported hydrodynamic size for the fully filled case starts to increase (B), reaching a value of ~1.6 × 10^−6^ m within a further 200 s (C), indicating that the underlying processes that cause the effects described in Fig. 1 *are not limited to extremely high molarities*: they are just much less severe.

The Fourier transform of the collected signal during the electrophoresis measurement should be of Lorentzian form^45^ with a half-width, half-maximum, HWHM = | ***q*** | ^2^*D*, where *D* is the self-diffusion coefficient, related to the particle diameter, 2.a, via the Stokes-Einstein equation,

1 $$a=\frac{{K}_{B}T}{6\pi \eta D}$$

Here, *η* is the sample viscosity, *K*_*B*_ is Boltzmann’s constant, *T* is the absolute temperature and ***q*** is the q-vector: an instrumental factor describing the wavelength of the light used to illuminate the sample, the angle at which the scattered light is collected with respect to the collimated light source and the refractive index of the sample dispersant. Estimation of the hydrodynamic diameter of the sample via the frequency distribution of the light collected *during* an electrophoresis measurement is fraught with uncertainty: an ill-defined sample viscosity due to Joule heating effects^45^ and the fact that the sample is also undergoing electrophoretic motion are notable factors. As shown in Fig. 2(d) and Supplementary Material, S4, an estimate of the particle size (±1 standard deviation) as falling between 75.5 nm and 244.8 nm is possible. Certainly, 200 nm falls well within this rather uncertain interval, however, we can further infer the preservation, or not, of the particle size during the electrophoresis measurement from the size and electrophoretic mobility data in combination. In Fig. 2(a) we have characterised the particle size has being (nominally) 200 nm using dynamic light scattering (DLS) in the absence of an applied electric field within the sample, before and after the electrophoresis measurement. Further, consider that the frequency spectra in Fig. 2(c) typically fall into two categories with the narrower distribution associated with the sample prior to the electrophoresis measurement. Therefore, since we know that the sample is undegraded at *t* = 0 we can infer that the narrower frequency spectra are indicative of the preservation of the starting particle size during the electrophoresis measurement. It follows then that the 200 mM KCl case in Fig. 2(a) and the 4.0 M KCl Diffusion barrier filled measurements (1–5) in Fig. 2(c) demonstrate that we can be sure that degraded material was not present in the optical detection volume during the electrophoresis measurements, even though, from Fig. 2(a), we know that degraded material *was* created and which subsequently diffused to the detection point, (B)– > (C), for the 200 mM case.

Similarly, looking at Fig. 2(c), the fully filled 4.0 M KCl electrophoresis measurements do not get to the end of the first measurement before the frequency spectrum is significantly affected by the presence of degraded material of widely varying hydrodynamic sizes. The degraded sample is polydisperse into the sub-millimetre scale by *visual* inspection of Fig. 1 (*top-row*) and much of the degraded fraction has settled to the bottom of the capillary, through the optical detection point broadening the frequency spectrum in Fig. 1(c) and making characterisation for particle size uninformative. It is in this sense that the diffusion barrier method is enabling for the routine, ensemble characterisation of electro-kinetic effects in high ionic strength dispersions.

Can we use these data to deduce more about the source of these effects? There are a number of effects that could conceivably contribute to the creation of the degraded fractions observed, during the application of the electric potential to the cell, from an otherwise stable polystyrene sample. Either individually or in combination these might include localised extreme Joule heating and high current flow in the dispersion and its interaction with the dispersed phase, including elevated current flow in the region at and around the particle/dispersant boundary or an interaction of the electrode surface, the current, the dispersant and the *presence* of the dispersed phase. It is out of scope to comment further in detail however, we are able to make additional deductions from the data to inform further consideration, including:-

- The arrival time of the degraded material at the measurement point in Fig. 2(a), region (B), is non-zero for both filled and unfilled cases. This indicates that the spoilt material is created remotely from the detection point and migrates there, over time.
- The degraded material arrived much later, after the end of the electrophoresis measurement, for the diffusion barrier filled, 200 mM KCl measurements.
- The material of the internal walls of the capillary is the same along its entire length including around the measurement point, (x) in Fig. 1. Therefore, if degraded material were created at the capillary wall or in the bulk of the fluid we would expect it to appear at the measurement point in similar timescales for fully filled cells and cells filled using the barrier method. In fact, since the only material discontinuity inside the capillary is the surface of the electrodes, then we propose that these degraded fractions are created in the vicinity of the electrode surface.
- This hypothesis is supported by the fact that the diffusion barrier filled 200 mM degraded sample took longer to arrive at the detection point than for the fully filled cell: the undegraded 200 nm polystyrene beads had to diffuse from the sample entry point, which coincides with the optical detection point, back to the electrode surfaces, before the subsequently degraded material could migrate back to the measurement point.
- the difference between the trending and catastrophic responses of the 200 mM and 4.0 M samples, respectively, indicates that the amount of degraded sample created is correlated with the current flow into the sample, during the electrophoresis measurement.

## Results and Discussion

In this section we use the diffusion barrier method and optimised measurement settings (Methods, below) to experimentally explore the dependency of electrophoretic mobility on *pH* and ionic strength and the transition from low to high ionic strength for a number of common aqueous dispersions.

Note that light scattering instruments measure the electrophoretic mobility of low particulate concentration, low ionic strength samples and calculate a zeta potential value from it, using the Henry equation^30^. This process is described briefly in the Theory and Calculations section, below, in order to make contact with the commonly referenced Debye length. However, for all experimental results we report the measured electrophoretic mobility, due to the ongoing development of the theoretical understanding of high molarity systems.

### Residual electrophoretic mobility

**Figure 3:**
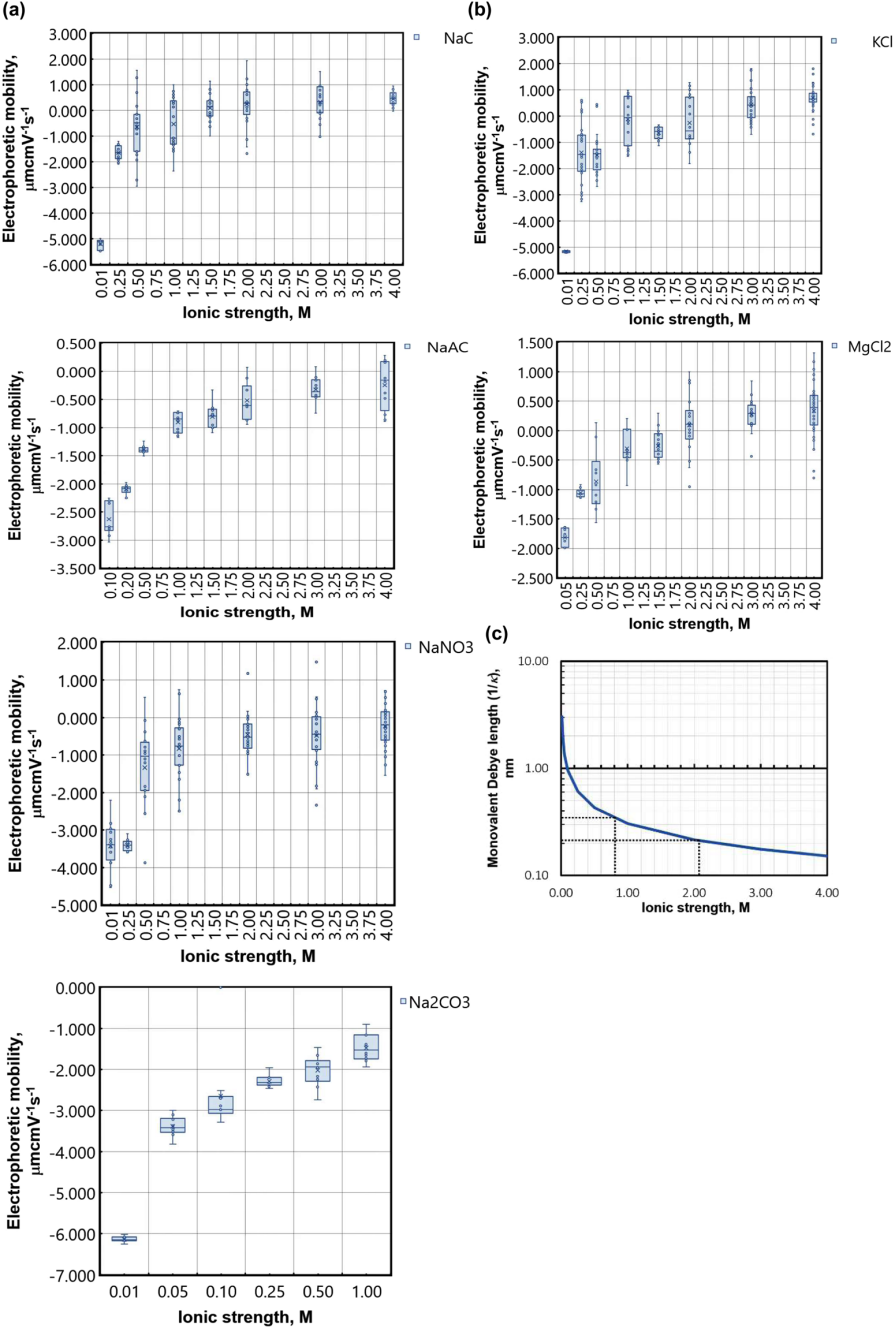
Asymptotic electrophoretic mobility with increasing ionic strength for (**a**) salts of Sodium and (**b**) salts of Chlorine, all adjusted to *pH* 7.0 using HCl and the appropriate Hydroxide salt (*x*OH). (**c**) The monovalent Debye length (Eq. 3) and solvated ion size estimate (dotted lines) – see main text. The mode is shown as a cross and the median as a line and the error bars represent ±1 standard deviation. All data in Supplementary Information, S1.

Figure 3 shows the data recorded using a diffusion barrier for 200 nm polystyrene beads in sodium chloride, sodium nitrate, sodium acetate, potassium chloride and magnesium chloride. The 200 nm particle size at the measured temperature of 25.0 °C ± 0.1 °C led to a diffusion rate away from the sample entry point slow enough to accommodate the entire measurement, including the thermal equilibration delay: Materials and Methods, below. From Fig. 3:-

- Note immediately that all salts were successfully measured over 20–30 data points per molarity, avoiding catastrophic effects such as outgassing or precipitating-out.
- The data were inspected for special causes using an Individual value control chart in Minitab v18.0 and typically no more than 2 data points were removed per molarity (4.0 M KCl, 4 points were removed) all on the basis of Nelson rule 1^46^: deviation greater than 3 standard deviations from the mean (Supplementary Material, S1). Therefore, no special causes were found in the data other than an occasional outlier. The instrument settings used were carefully optimised (Methods, below).
- A surprising theoretical prediction is that over-screening of the particle surface charge may occur when ion-ion pair correlations^36,39^ become important with increasing ionic strength, resulting in an apparent particle surface charge inversion and a reversal of the sign of the electrophoretic mobility. This is clearly demonstrated in Fig. 3 for salts of chlorine but not of -acetate, -nitrate or -carbonate.
- Particle aggregation is known to occur quickly in high ionic strengths or for weakly charged particles and to slow down for low ionic strength systems^42^ and the transition from high (magnitude) electrophoretic mobilities at low ionic strength to low electrophoretic mobilities at high ionic strength in Fig. 3, supports this observation.
- The asymptotic electrophoretic mobility in the saturated limit is postulated to follow a Hofmeister series dependency^38,40^ with the value of the asymptote dependent on the identity of the ionic species present. Additionally, anions have been found to have a larger effect than cationic species^47^. Anions specific to this study are ordered as C_2_H_3_O_2_^−2^ (acetate) > Cl^−1^ > NO_3_^−^, where the order is based on increasing ion hydration^48^. Similarly, the cationic species studied should follow the rule, K^+^ > Na^+^ > Mg^2+^.
  Referring to Supplementary Data S2, the 4.0 M data in Fig. 3 for sodium chloride (n = 19) and magnesium chloride (n = 52) are normally distributed (*p* = 0.05), but the data for potassium chloride (n = 45) are not (*p* = 0.05). The medians for the data at 4.0 M are +0.80 μmcmV^−1^s^−1^ for potassium chloride, +0.46 μmcmV^−1^s^−1^ for sodium chloride and +0.35 μmcmV^−1^s^−1^ for magnesium chloride. Using a Mann-Whitney test with non-equal variance and *p* = 0.05, it is not possible to reject the null hypothesis that the median values for sodium chloride and magnesium chloride at 3.0 M and 4.0 M are the same. The same null hypothesis for 3.0 M and 4.0 M potassium chloride is rejected, however, the median value at 4.0 M for potassium chloride is already in excess of the values for sodium chloride and magnesium chloride. We can therefore state that the cationic Hofmeister series is reproduced (*p* = 0.05).
  The anionic case is more complex as the median electrophoretic mobilities at 4.0 M ionic strength are −0.23 μmcmV^−1^s^−1^ for sodium nitrate and −0.25 μmcmV^−1^s^−1^ for sodium acetate. However, both of these points are significantly different to the value for sodium chloride (*p* = 0.05) by (+0.46 μmcmV^−1^s^−1^) – (−0.23 μmcmV^−1^s^−1^) = 0.69 μmcmV^−1^s^−1^. So, we have C_2_H_3_O_2_^−2^ < Cl^−^ and NO_3_^−^ < Cl^−^, but sodium nitrate and sodium acetate are indistinguishable from each other.
  Therefore, whilst we note that the difference between sodium chloride and sodium acetate and also sodium chloride and sodium nitrate is higher for the anionic case as expected^48^, sodium nitrate and sodium acetate are not resolved and further, both asymptote to a value less than, not higher than, the value for sodium chloride.
- Finally note that we were unable to source carbonic acid at a high enough concentration to adjust the *pH* to 7.0 for molarities beyond 1.00 M Na_2_CO_3_ without significantly diluting the sodium ion concentration, but since it is a commonly found compound in water treatment and food manufacturing, the data are reported for information.

### Contact with the gouy-chapman model: the debye length

We are advised that the classical EDL should ‘collapse onto the particle surface’ as the ionic strength of the dispersant approaches 1.00 M and the Debye length approaches the solvated ion size^49^. In this section we compare the Debye length from the classical Gouy-Chapman model to the electrophoretic mobility results in Fig. 3. Marcus^50^ uses molecular dynamics, Monte Carlo simulations and x-ray diffraction measurements to characterise a wide range of ions, providing an estimate of the solvated ion radius out to the first hydration shell. Considerable detail is required for each of these studies, but uncertainties most pertinent to this work, include:

- The calculations are made out to the first solvation shell only.
- An approximate value of the solvated water ‘radius’ is taken as 0.138 nm ± 0.000 nm. This is the water ice value and is used in preference to the room temperature value of 0.143 nm ± 0.000 nm assuming that the ‘immobilised’ value better represents the solvated value.
- The intermolecular water distance in the bulk of the dispersant, outside of the hydrated layer, is assumed to be that of pure water since the water molecules out-number those of the ions by an order of magnitude.

Under these assumptions the solvated ionic radius for the species considered in this work can be taken to lie in the approximate interval [0.21, 0.35] and this is shown as the dotted lines on Fig. 3(c). Projecting this region onto the Debye length curve, derived from the Gouy-Chapman model^30^ should then provide an estimate of the range of ionic strengths over which the EDL^30^ can be assumed to ‘collapse’ onto the particle surface and beyond which ‘other’ means for the creation of zeta potential should dominate. We note that this region, ~0.8 M to ~2.0 M agrees quantitively, relatively well with the region of steadily reducing 2^nd^ derivative (δ^2^mobility/δ ionic strength^2^) from the experimental data in Fig. 3, that appears to sit between the classical (high 1^st^ derivative, δmobility/δ ionic strength) and structured water layer (~zero 1^st^ derivative) models. It is not possible to rule out a discontinuous transition between these two regimes of magnitude smaller than the uncertainties shown in Fig. 3, however, a ‘hard stop’ at 1.0 M for the classical regime is not supported by these data: consider for example that the electrophoretic mobilities at 1.00 M and 1.50 M for magnesium chloride are not statistically separable.

### The transition from low to high molarity: desensitisation to *pH*

In this section we explore the transition from the low to high ionic strength regimes using titrations of *pH* and molarity.

It is important to note that despite a good quantitative and qualitative agreement with theory it is not possible to tell from Fig. 3 whether the dependency of electrophoretic mobility on ionic strength is due to a specific interaction of hydrated counter ions with the hydrophilic surface of the polystyrene beads^42,51,52^ or a selective process within the structured water layer around the particle in high ionic strengths^36,39^, some other effect or a combination of effects. However, we would expect the titration of *pH* to primarily affect the charge at the particle surface and this may allow us to be more specific.

**Figure 4:**
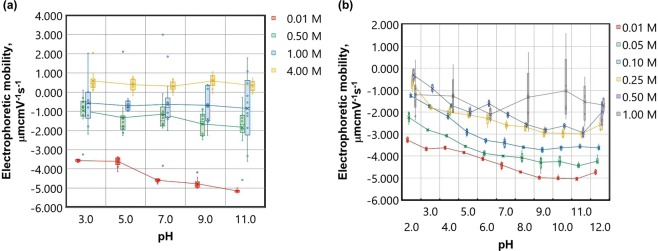
*pH* titrations of 200 nm polystyrene beads with increasing salt concentration for aqueous solutions of (**a**) NaCl and (**b**) NaNO_3_. The mode is shown as a cross and the median as a line and the error bars represent ±1 standard deviation.

The data in Fig. 4 are from titrations of *pH* in aqueous solutions of NaCl and NaNO_3_. The lower molarities of the NaNO_3_ data [0.01 M, 0.05 M] were recorded without a diffusion barrier using an autotitrator, whereas the 1.0 M NaNO_3_ and all of the NaCl data were measured using the diffusion barrier method. A number of features are apparent: Firstly, note that in the high ionic strength cases of 1.00 M and 4.00 M NaCl and 1.00 M NaNO_3_, the residual electrophoretic mobility has become invariant with *pH*. The relationship between *pH* and electrophoretic mobility is based on the ionisation of acidic or basic groups at the particle-fluid boundary, by the adsorption of OH^−^ or H^+^ to give a more negatively or more positively charged surface, respectively. An excess of counter-ions from the surrounding fluid phase then builds up around the particle and the classical EDL and a *pH* dependent zeta potential results. The observed desensitisation to *pH* with increasing ionic strength provides strong evidence that the residual zeta potential towards and at saturated molarities is not created directly due to charge on the particle-fluid boundary^36,39^.

Also, we note that the absence of an isoelectric point in the high ionic strength regime, between *pH* 2.0 and *pH* 11.0, may have important implications for the chemical removal of nano- and micro-particle contaminants from environmental, physiological and industrial systems. Also, towards, *pH* 2.00, the 0.50 M curves for both NaCl and NaNO_3_ approach that of the 1.00 M curve, indicating potential savings for industrial processes that operate in the acidic regime.

## Methods

### Materials

The 200 nm came from Thermo Scientific, UK, (3200 A), all other chemicals were supplied by Sigma-Aldrich (UK) to ACS grade (>95%). The water used throughout was deionised (18.2 MΩ, Millipore, UK), filtered to 200 nm for rinsing and 20 nm for sample preparation using Whatman Anotop filters. The pipette tips used to implement the Diffusion Barrier method were Costar ™ gel-loading tips (Sigma-Aldrich, UK, CLS4853).

### Apparatus

All batch data were recorded using a Zetasizer Ultra with autotitrated data recorded using a Zetasizer Pro and an MPT-3 Multi-purpose Titrator and Autodegasser in disposable folded capillary cells (DTS1070/80), for both cases (Malvern Panalytical Ltd, UK). The zeta potential was measured in monomodal, constant-current mode, using phase analysis light scattering (PALS) to characterise electrophoretic mobility from a fast-field reversal, described in more detail in *Instrument settings*, below.

### Cuvette loading procedure

For all measurements, each cell was rinsed through thoroughly with methanol and then copious amounts of 200 nm filtered DI water (18.2 MΩ). One cell per aliquot was used throughout to avoid cross-contamination. All data were recorded at 25.0 °C, unless otherwise stated, the cuvette was thermalized in the instrument prior to filling with the sample and then the sample allowed to thermalize with the cell and instrument for 120 s, prior to the beginning of the measurement. This delay was titrated up to this value until no special cases were measured in the first few measurements of each aliquot, such as bulk thermal trending or significant changes in the conductivity of the sample.

For all batch measurements the cuvettes were loaded using the diffusion barrier method^43^, Fig. 1. Typically, 0.8 mL of each salt solution tested was used to fill the cuvette and a gel loading tip was then used to place a 100 µL aliquot of a 0.2% 200 nm polystyrene beads decanted from the manufacturers bottle, dispersed in the same bulk salt solution under test to avoid dilution, at the optical detection point (***x***, Fig. (1)) of the instrument at the bottom of the cell. In this way, the dispersed phase remains in electrical contact with the instrument but is physically isolated from the electrode surface. The electrophoresis measurement completes long before the dispersed phase is able to reach the electrodes by diffusion or electro-osmotic motion, within the cell.

### Sample preparation

**Figure 5:**
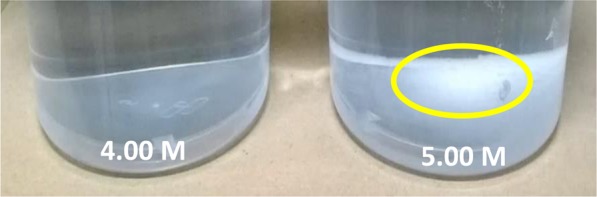
5.00 M KCl incompletely dissolved at 20.0 °C.

Solutions of sodium chloride (Thermo Fisher Scientific) potassium chloride, magnesium chloride, sodium nitrate, sodium acetate and sodium carbonate (Sigma-Aldrich) were prepared to 4.00 M and then filtered through a 200 nm Whatman Anotop ^TM^ filter. They were then further diluted in series to make up solutions of 2.00 M, 1.00 M, 0.50 M, 0.25 M, 0.10 M, 0.05 M and 0.01 M and the *pH* adjusted to 7.00. A practical saturation limit was found of ~4.00 M for KCl and NaCl at 20.0 Celsius (sea-level), with undissolved salt still visible at the bottom of the preparation beaker after 24 hrs of gentle agitation of a 5.00 M solution, on a laboratory roller, Fig. 5.

For the diffusion barrier measurements, typically, 100 μl of polystyrene beads as received from the manufacturer was added to 10.0 mL of salt solution using a hand micro-pipette via reverse addition to reduce the impact of the change in conditions in the higher concentration solutions and the *pH* adjusted with HCl and the corresponding base: NaOH in NaCl_2_, KOH in KCl, etc. For the autotitrated measurements, samples of NaNO_3_ were prepared to concentrations of 0.05, 0.10, 0.25, and 0.50 M, and *pH* adjusted to the titration starting point of *pH* 12, using NaOH. For all concentrations, 10 mL aliquots were added to the 200 nm polystyrene beads to make 1% *v*/*v* mixtures.

### Instrument settings

The samples characterised were not low concentration and the Zetasizer Pro and Ultra were used interchangeably as they are essentially equivalent for this application. For the batch zeta potential measurements, a Zetasizer Ultra was used with a 633 nm HeNe gas laser and zeta potential analysis version 7.1, in the ZS XPLORER software. A 1.0 kHz modulator frequency was used with the optical attenuation set to automatic in the instrument software standard operating procedure (SOP). The instrument measures the count-rate from the sample and auto-attenuates the laser until the ratio of the sample to reference beam scattering is 1:8^53^. The conductivity of the sample is then characterised using a low field strength to minimally perturb the sample and then 10 measurements of 30, 1 s sub-runs were performed at 25 °C with a 180 s pause between each measurement, to allow joule heating of the sample to dissipate^45^.

**Figure 6:**
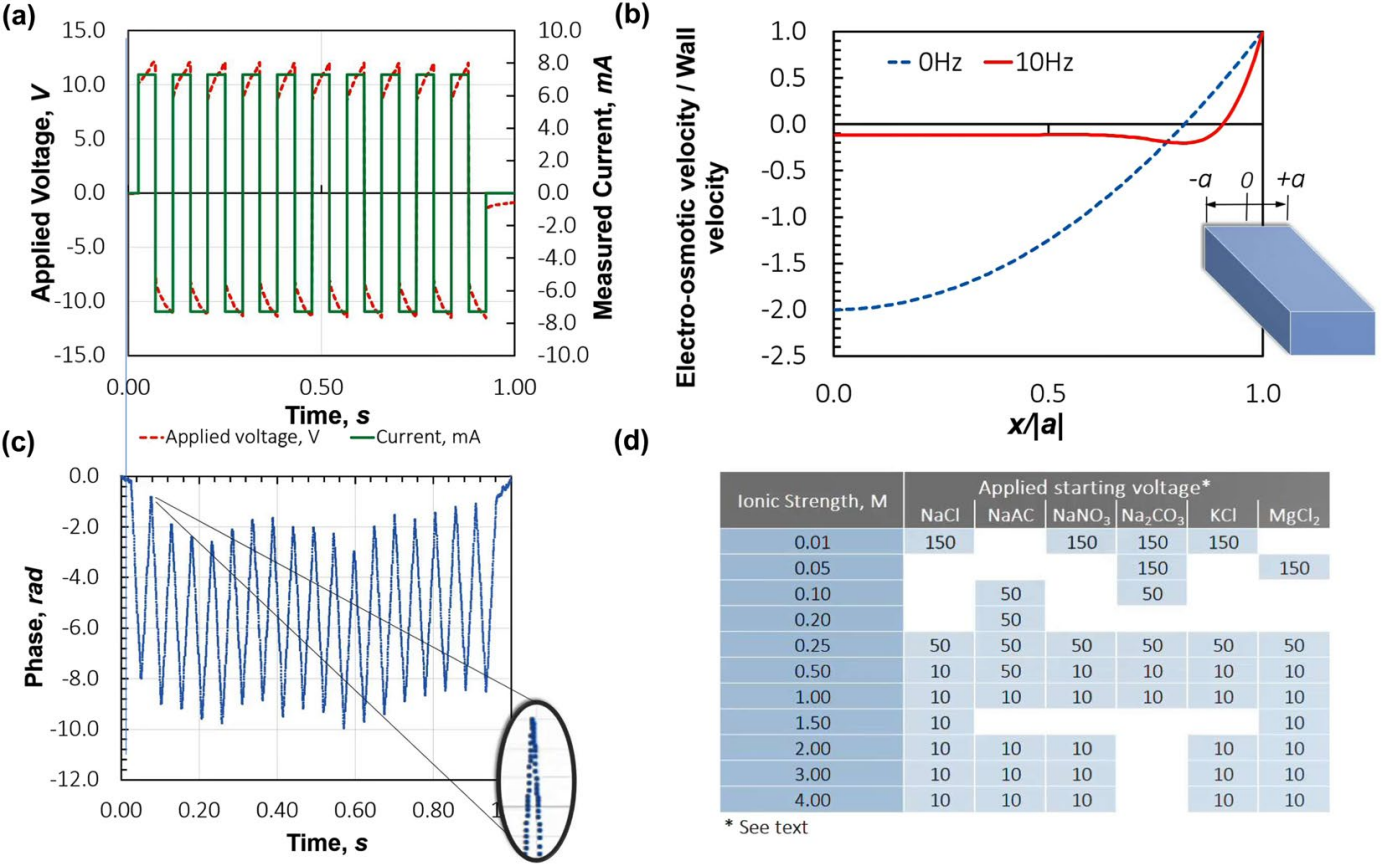
Measurement settings – (**a**) fast field reversal cell drive in constant current mode, (**b**) fast-field reversal suppresses electro-osmosis at the centre of cell, where the optical measurement point resides and (**c**) the resulting phase plot from which the electrophoretic mobility is estimated, (**d**) the starting potential applied across the cell, for each molarity.

The applied electric potential reversal to the cell is shown in Fig. 6(a). Schatzel *et al*.^54^ present a theory describing the suppression of electro-osmotic flow created at the walls of a capillary of half-width, *a*, under the application of an electric field along the capillary, towards the capillary centre and, in this case, coincident with the optical measurement point in the capillary (*x*/*a* = 0), for an oscillating field of a particular frequency. The field reversal results, using this theory, are shown in Fig. 6(b) for the folded capillary cell. Note that the electro-osmotic component falls to nearly zero at the cell centre at 10 Hz and in practice the residual value falls into the noise of the measurement and is not commonly detected, even at low mobilities. All measurements in this work were recorded with automatic field reversal settings described in Fig. 6(d), which the software selects based on the conductivity of the sample.

It is out of scope to discuss phase analysis light scattering (PALS) in detail here as it is described elsewhere^55–57^. For our purposes, note that a PALS measurement yields an estimate of the phase shift of the light scattered by the particles in the sample undergoing linear motion in the applied electric field, with reference to the collimated, illuminating light beam: usually a laser. Many such PALS measurements recorded sequentially result in a cumulative phase shift within each field reversal, increasing or decreasing in value depending the direction of the applied field and the sign of the electrophoretic mobility of the dispersed phase. This is made clear in Fig. 6(c), where each point in the phase plot is an individual measurement, *φ*_*i*_, over a small time interval, of the phase of the light scattered from the sample particles moving under the applied electric field. In the case shown, the phase decreases linearly over the positive half of the field reversal and then changes direction in response to the field reversal of the opposite sign. Consecutive samples of *φ*_*i*_ then yield a phase change per unit time, *ϕ = φ(t)*, to which an electrophoretic mobility, *μ* (μmcmV^−1^s^−1^) in a *known* field strength, *E (*Vcm^−1^), can be assigned via

2 $$\phi =|{\boldsymbol{q}}|\mu E$$

Under the application of each field reversal, solvated ions (as well as the dispersed phase) migrate towards their respective counter-electrodes. This time-resolved re-distribution of charge results in a counter electric field that opposes the applied field. Subsequently, the current falls off approximately exponentially over each field reversal, meaning that for high molarities the field strength is poorly estimated from the applied potential. The field strength, *E*, (Eq. 2) at the measurement point is the product of the conductivity of the sample, the measured current & a constant cell geometry factor and a non-constant current over the field reversal leads to significant uncertainty in the estimate of *E* at the measurement point (*x*, Fig. 1) and also creates curvature in the measured phase plot due to the varying field strength, increasing the uncertainty in *μ* further, via an additional uncertainty in the estimate of *ϕ*, in Eq. 2. This problem is mitigated by titrating the applied electric potential over each field reversal, from a fixed starting value, to keep the resulting current constant, Fig. 6(a,d). This leads to a constant current over the entirety of each field reversal, which significantly reduces the uncertainty in the estimates of both the current and the average phase and therefore, in *E* and *ϕ*, respectively.

The starting voltages were titrated up and the set values are derived on the basis that the maximisation of the field strengths will minimise the uncertainty in the electrophoretic mobility, but below the point at which statistically significant changes in the conductivity of the dispersant is measured. These ideas are explored in detail in Corbett & Jack^46^. For this work, please note the starting voltage values in Fig. 6(d) and that instrument SOP’s are available in Excel spreadsheet form in Supplementary Material S4.

The *pH* autotitration measurements were completed using an MPT-3 Multi-purpose autotitrator, Autodegasser and a Zetasizer Pro in conjunction with clean DTS1070 cells. The titrations ran from *pH* 12–2 with step sizes of *pH* 0.5. At each *pH* point, 5 monomodal zeta potential measurements were made, with a maximum of 30 runs per measurement, and a 60 s pause between each repeat measurement. All titrations were completed using titrants of 0.250 M and 0.025 M HCl and conducted at 21.0 ± 0.1 degrees Celsius, to match the laboratory ambient temperature.

### Theory and calculations

Micro-electrophoresis instruments measure the electrophoretic mobility, *U*_*E*_ of the dispersed phase, under the application of an external electric field. The zeta potential, the electric potential at the hydrodynamic slipping plane within the EDL, is typically calculated from the measured electrophoretic mobility via the Henry equation,

3 $${U}_{E}=\frac{2\varepsilon \zeta f(\kappa a)}{3\eta }$$

where, ζ is the zeta potential, *ε* is the dielectric constant, *η* the viscosity, *f*(*κa*) is Henry’s function, *a* is the particle radius and the quantity *κ*^−1^, is the Debye length describing the extent of the EDL beyond the particle radius, *a*. The Debye length *κ*^−1^ can be calculated for a monovalent electrolyte using Hunter 1981^30^,

4 $$\kappa ={\left\{\frac{{\sum }_{i=1}^{N}{e}^{2}{z}_{i}^{2}{n}_{i}^{2}}{{\varepsilon }_{rs}{\varepsilon }_{0}kT}\right\}}^{1/2}$$

where, *e* is the elementary charge*, z*_*i*,_*n*_*i*_, charge number and number concentration of ion, *i*, *ε*_*rs*_ is the relative permittivity of the electrolyte solution, *ε*_*o*_ the permittivity of free space, *k* is Boltzmann’s constant and *T* the absolute temperature, all over *N* ions in solution, in total. At a fixed temperature of 25.0 °C, this reduces to

5 $${\kappa }^{-1}(nm)=\frac{0.304}{\sqrt{I(M)}}$$

and so, phenomenologically, as the ionic strength of the surrounding dispersant is increased, the double layer is suppressed in size.

We make contact with these quantities where appropriate, however, significant uncertainties may exist particularly in the values of the dielectric constant, *ε*, the viscosity, *η* and the Henry function, *f(κa*) as the ionic strength of the dispersant increases. Therefore, we avoid the conversion to zeta potential and report the directly measured electrophoretic mobility.

## Conclusions

Using a standard laboratory ensemble light scattering instrument we find that the diffusion barrier method, which isolates the dispersed phase from the electrodes, prevents the catastrophic sample degradation, outgassing and precipitation observed when the particles are in contact with the electrode surface. This tool enables the routine, ensemble characterisation of high ionic strength dispersions in small sample volumes and short measurement times.

We have reproduced a number of electro-kinetic effects predicted by theory to exist at and approaching saturated ionic conditions in aqueous dispersions, including asymptotic and non-zero electrophoretic mobility, an ion-specific value of the asymptote and charge inversion. These tools should help accelerate the ongoing development of a complete electrokinetic theory of elevated ionic strength systems and their application to important contemporary problems in the environmental sciences, pharmacology and industrial and pharmaceutical manufacturing.

## Supplementary information

Supplementary information.

.Supplementary information2

Supplementary information3

Supplementary information4

Supplementary information5
