# Supplementary material for: Routine, ensemble characterisation of electrophoretic mobility in high and saturated ionic dispersions
Source: Sci Rep. 2020 Mar 13;10:4628. doi: 10.1038/s41598-020-61624-9 (PMC7070055; doi:10.1038/s41598-020-61624-9)
Supplement: Supplementary file 1 — Supplementary information. [file 41598_2020_61624_MOESM1_ESM.pdf]

Routine, ensemble characterisation of electrophoretic mobility in high and saturated ionic dispersions

Jake Austin[1], Diogo Fernandes[1], Matthew J A Ruszala[1],Natalie Hill[1] & Jason Corbett[1]
1 Nanotechnology group of Malvern Panalytical Ltd, Grovewood Road, Malvern, WR14 1XZ. Correspondence and requests for materials should be addressed to J.C. (email: jason.corbett@malvernpanalytical.com).

Supplementary material 1
Raw data, statistical outlier analysis and boxplots for Figure 3

Supplementary material 2
Normality tests and Mann-Whitney test for figure 3

Supplementary material 3
Standard operating procedures for the ZS Explorer software for the Zetasizer Ultra (available on request) for KCl, MgCl2, NaCl, NaNO3. Data for NaAC and NaCO3 were recorded using the NaCl SOP, but with the sample name changed accordingly.

Supplementary material 4
Frequency to size estimate for figure 2
